# A Mucosal Adenovirus Prime/Systemic Envelope Boost Vaccine Regimen Elicits Responses in Cervicovaginal and Alveolar Macrophages of Rhesus Macaques Associated With Delayed SIV Acquisition and B Cell Help

**DOI:** 10.3389/fimmu.2020.571804

**Published:** 2020-09-30

**Authors:** Ruth Hunegnaw, Sabrina Helmold Hait, Gospel Enyindah-Asonye, Mohammad Arif Rahman, Eun-Ju Ko, Christopher J. Hogge, Tanya Hoang, Marjorie Robert-Guroff

**Affiliations:** Immune Biology of Retroviral Infection Section, Vaccine Branch, National Cancer Institute, National Institutes of Health, Bethesda, MD, United States

**Keywords:** cervicovaginal macrophage, alveolar macrophage, simian immunodeficiency virus, B cell help, rhesus macaques, FcγRIII, replicating adenovirus recombinant vaccine

## Abstract

Vaccine strategies targeting the mucosal portal of entry may prevent HIV acquisition and systemic infection. Macrophages in cervicovaginal compartments are one of the first cell types to encounter virus upon vaginal exposure. Their activation can lead to recruitment of additional macrophages and CD4^+^ T-cells susceptible to viral infection. However, they are also critical in providing early protection against invading pathogens. Therefore, understanding their response to immunization is important for vaccine design. We immunized rhesus macaques twice mucosally with replicating adenovirus (Ad) SIV recombinants, followed by two intramuscular boosts with SIV gp120 protein. Macaques were subsequently challenged intravaginally with repeated low doses of SIV_mac251_. Using flow cytometry, we evaluated responses of cervicovaginal macrophages (CVM) and alveolar macrophages (AM) in bronchoalveolar lavage as initial immunization was to the upper respiratory tract. The frequency of CVM increased over the course of immunization; however, CCR5 expression significantly decreased. Significantly increased expression of the chemokines CCL3 (p < 0.01), CCL4, CCL5, and CXCL8 (p < 0.0001 for all) on CVM was seen post-1^st^ Ad but their expression significantly decreased post-2^nd^ boost. CD4^+^ T-cell frequency in the cervical mucosa remained unchanged. CVM FcγRIII expression was significantly increased at all time points post-immunization compared to naïve animals. FcγRIII expression post-2^nd^ Ad positively correlated with the number of challenges needed for infection (r = 0.68; p = 0.0051). Vaccination increased AM FcγRIII expression which post-2^nd^ boost correlated with antibody-dependent phagocytosis. Activation of AMs was evident by increased expression of CD40 and CD80 post-2^nd^ Ad compared to naïve macaques. APRIL expression also significantly increased post-2^nd^ Ad and correlated with B cell frequency in bronchoalveolar lavage (BAL) (r = 0.73; p = 0.0019) and total IgG in BAL-fluid (r = 0.53; p = 0.047). B cells cultured with SIV gp120-stimulated AM supernatant from vaccinated macaques exhibited significant increases in B cell activation markers CD38 and CD69 compared to B cells cultured alone or with AM supernatant from unvaccinated macaques. Overall, the vaccine regimen did not induce recruitment of susceptible cells to the vaginal mucosa but increased CVM FcγRIII expression which correlated with delayed SIV acquisition. Further, immunization induced expression of AM cytokines, including those associated with providing B cell help.

## Introduction

Mucosal surfaces including the lungs, gut, sensory, and reproductive organs are permeable due to their physiological function. This makes the areas vulnerable to pathogens and mucosal immunity crucial for preventing invasion by infectious agents like human immunodeficiency virus (HIV). The vaginal and rectal mucosa are known to be the primary sites of HIV transmission (1). After vaginal exposure, once the endothelial barrier is crossed, a local increase of chemokine expression by the cervical epithelium leads to recruitment of CCR6^+^ plasmacytoid dendritic cells (pDC) and macrophages expressing chemokine receptors CCR5, CCR6, and CXCR2 (2, 3). These cells create an environment rich in chemokines such as CCL3 (MIP-1a), CCL4 (MIP-1B), CCL5 (RANTES), CXCL8 (IL-8), and CXCL10 (IP-10), which lead to the recruitment of susceptible CD4^+^ T cells (4). Indeed, recent studies in rhesus macaques have shown that CD4^+^ T cells, specifically of the Th17-lineage, are primary target cells of simian immunodeficiency virus (SIV) (5). When infected, these cells form the small founder population that initially expands in the cervicovaginal mucosa leading to dissemination and systemic infection. Therefore, vaccines that can elicit strong mucosal immunity while preventing access to target cells at the site of exposure are crucial for successful protection against HIV/SIV.

In this study, we used a mucosally administered replication competent recombinant adenovirus type 5 host range mutant (Ad5hr) expressing SIV *env*, *nef*, and *gag* genes coupled with envelope systemic boosting in order to generate long-lasting immunity. Ad5 is no longer being pursued as an HIV vaccine candidate due to previous failures in clinical trials, however numerous other Ad-vectored approaches are being explored (6), including replicating adenovirus (Ad) vectors (7, 8). Replicating vaccines are highly effective and provide long-lasting immunity (9). However, Ad are severely host-range restricted, permissive for humans but not rhesus macaques. In order to investigate replicating Ad vaccines in the SIV/rhesus macaque system, we have used the Ad5hr vector (10) as a model since it replicates in rhesus macaque cells (11) and has been shown to result in viral shedding in mucosal compartments post-intranasal/oral priming of rhesus macaques, resulting in efficient induction of protective immune responses (12, 13).

We have previously reported that immunization of rhesus macaques with our replicating Ad5hr-recombinant approach affects many cells of the innate immune system. MAIT cells can be stimulated by vaccination leading to enhanced B cell responses (14). Replication-competent adenovirus-SIV recombinants induced neutrophil activation, B cell help markers, greater ability to generate reactive oxygen species, and greater potential to provide B cell help (15). Mucosal replicating Ad-SIV immunization elicited functional activation of rectal DCs with the potential to induce local and systemic antigen-specific immune responses (16). Studies have also shown that intranasal/intratracheal Ad administration can target alveolar macrophages (AM) found in bronchoalveolar lavage (BAL) (9). This encounter can lead to immune responses that may be beneficial for vaccine outcome. Indeed, it has been reported that AMs can induce adaptive immune responses not only by processing antigen and presenting it to effector T-cells but also by transporting antigen to the lung draining lymph nodes (dLN) prior to migration of pathogen-induced lung dendritic cells (DC) (17). AMs in the dLN were localized primarily in B cell regions indicating a possible interaction between alveolar macrophages (AM) carrying antigen and B cells (17). An indirect effect of AMs on B cell responses is also possible due to expression of cytokines like BAFF and APRIL, key promoters of B cell activation and expansion. In mice and humans, BAFF and/or APRIL expression by AM has been shown in the context of TLR-7 signaling and pulmonary disease settings (18, 19).

Given that AMs are one of the first cells encountered following priming with the Ad5hr recombinant vaccine, it is important to understand their activation and function following vaccination. Further, macrophages found in the cervicovaginal compartment are also one of the first cell types to encounter the virus during vaginal challenge post-immunization. As their response to virus exposure has been shown to potentially increase recruitment of susceptible cells (4), understanding their dynamics and chemokine profile after immunization is important for vaccine design.

## Materials and Methods

### Animals, Immunization Schedule, and Sample Collection

**Figure 1:**
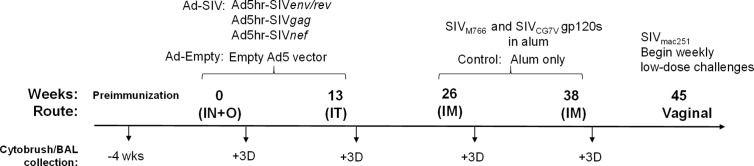
Scheme showing the immunization and sample collection schedule. As detailed in *Materials and Methods*, adenoviral immunizations were administered intranasally (IN) plus orally (O) and intratracheally (IT) at weeks 0 and 13, respectively. Intramuscular (IM) boosts with SIV gp120 proteins were given at weeks 26 and 38. Macaques were challenged vaginally with repeated low-doses of SIV_mac251_ beginning at week 45. Cytobrush and BAL samples were taken prior to immunization and 3 days after each immunization.

As previously reported (20) female rhesus macaques were immunized at week 0 (intranasally and orally) and week 13 (intratracheally) with replicating Ad5hr recombinants expressing SIV_smH4_*env/rev*, SIV_239_*gag*, and SIV_239_*nef* (*n* = 38, Ad-SIV) at a dose of 5 × 10^8^ plaque forming units/recombinant/route/macaque or with Ad5hr empty vector (*n* = 22, Ad-empty) at a dose equivalent to the Ad-SIV recombinants administered. The intranasal administration (250 μl/nostril) was given dropwise from a syringe. The oral administration (500 μl) was given by gastric tube to the stomach following injection of 2 ml sodium bicarbonate solution (1.5 g/100 ml H_2_O). This was followed by 1 to 5 ml of H_2_O or phosphate-buffered saline (PBS). The intratracheal administration to the trachea (500 μl) was performed using an endotracheal tube with a smaller flexible ureteral catheter insert and followed by a small volume of air. We have shown the replicating Ad recombinants initially target macrophages in bronchoalveolar lavage (BAL) fluid and rectal tissue, and later extend to myeloid dendritic cells in BAL fluid with persistent expression in the rectal mucosa at least 25 weeks after the last Ad immunization (21). As reported, similar biodistribution patterns are seen when the replicating Ad recombinants are administered sublingually, vaginally, or rectally. At weeks 26 and 38, recombinant SIV_M766_ and SIV_CG7V_ gp120 protein boosts (200 μg each protein/dose/macaque) in 2 ml final volume containing 1% alum hydroxide (Alhydrogel; InvivoGen) were given intramuscularly to the Ad-SIV group (n = 38) while alum only was given to the Ad-empty group (n = 22). Animals were challenged intravaginally beginning at week 45 with repeated weekly low-doses (800 tissue culture infectious doses) of the titered SIV_mac251_ 2010 day 8 stock originally prepared by Dr. R. C. Desrosiers and provided by Dr. Nancy Miller, DAIDS, NIAID. Up to 15 challenges were administered until infection was confirmed by plasma viral loads of ≥ 50 SIV RNA copies/ml plasma as assessed by the droplet digital PCR method (H. K. Chung, J. Narola, H. Babbar, M. Naseri, N. Richardson, R. Pal, and T. Fouts, manuscript in preparation). In additional analyses reported elsewhere (20), 20 of the Ad-SIV and 12 of the Ad-empty macaques were administered a 0.8% formulation of SAMT-247 (S-acyl-2-mercaptobenzamide thioester) microbicide gel (22) vaginally 3 h prior to each challenge. Here, macaques which received this microbicide were excluded from analyses involving post-infection outcomes. All rhesus macaques were maintained at the National Cancer Institute (NCI) animal facility under the guidelines of the Association for the Assessment and Accreditation of Laboratory Animal Care. The protocol (VB-027) and procedures were approved by the NCI Animal Care and Use Committee prior to initiation of the study. Immunization and sample collection schedules are shown in **Figure 1**. Two cytobrush samples from each macaque were collected in conical tubes containing 5 ml Roswell Park Memorial Institute Medium (RPMI) before and at day 3 after each immunization. Cells were detached from the brushes by scraping the tip against the opening of the conical tube. Samples were then centrifuged at 1500 RPM, resuspended in PBS, and filtered through a 0.70 µm filter. BAL samples were obtained using standard techniques (23, 24). Briefly, rhesus macaques were anesthetized, and an endotracheal tube was inserted through which sterile saline solution (10 ml/kg) was instilled. Suction was applied to recover the instilled fluid and the lung lavage was collected in sterile conical tubes. Cells were pelleted by centrifuging at 1600 RPM for 10 min at 4°C and washed in 25 ml cold PBS. Centrifugation was repeated, and cells were resuspended for counting. Cells showed ~90% viability as determined by trypan blue staining.

### Flow Cytometry

Antibodies used for staining BAL cells and cervicovaginal macrophages (CVM) are detailed in **Table 1**. For surface staining, antibodies were added to cells and incubated for 25 min at room temperature (RT). Subsequently, for intracellular staining, cells were washed with PBS prior to resuspending in BD Cytofix/Cytoperm™ and incubating for 15 min at RT. Cells were then washed with BD Permwash, and incubated with intracellular staining antibodies for 25 min at RT. Finally, cells were washed with PBS and staining data were acquired using the 5 laser BD FACSymphony (BD Biosciences, San Jose, CA). Fluorescence minus one (FMO) and isotype controls were used to confirm the phenotype and cytokine expression. Data analyses were performed with FlowJo (version 10.5.3, Tree Star, Inc Ashland, OR) software.

**Table 1:** Antibodies used for flow cytometry.

Assay	Marker	Clone	Fluorochrome	Source	Application
BAL B Cells	CD19	J3-119	PC5.5	Beckman Coulter	Surface
	CD20	2H7	BV650	BD Biosciences	Surface
AM	CD80	L307.4	BV421	BD Biosciences	Surface
	CD86	IT2.2	PE	BD Biosciences	Surface
	CD40	5C3	APC-R700	BD Biosciences	Surface
	CD206	19.2	PE-Cy7	Thermo Fisher	Surface
	CD163	GH1/61	BV510	BioLegend	Surface
	APRIL	Polyclonal	FITC	MyBioSource	ICS
	BAFF	1D6	APC	Thermo Fisher	ICS
AM and CVM (common antibodies used)	CD45	D058-1283	BV786	BD Biosciences	Surface
	CD4	L200	BV711	BD Biosciences	Surface
	CD3	SP34-2	BUV395	BD Biosciences	Surface
	HLA-DR	L243	PE-Cy5	BioLegend	Surface
	CD11b	ICRF44	APC-Cy7	Thermo Fisher	Surface
	FcγRIII	3G8	BUV496	BD Biosciences	Surface
	FcγRI	10.1	BUV737	BD Biosciences	Surface
	Live/dead	–	Blue Dye	Thermo Fisher	Surface
CVM	CCR6	11A9	APC-R700	BD Biosciences	Surface
	CCR5	2D7	BV605	BD Biosciences	Surface
	CXCR2	6C6	BV650	BD Biosciences	Surface
	CD68	Y1/82A	PE-Texas Red	Thermo Fisher	ICS
	RANTES	2D5	BB515	BD Biosciences	ICS
	MIP-1⍺	11A3	APC	BD Biosciences	ICS
	MIP-1β	D21-1351	BV421	BD Biosciences	ICS
	CXCL10	J034D6	PE	BD Biosciences	ICS
	IL-8	G256-8	BV510	BD Biosciences	ICS
Cervical CD4^+^ T Cells	CD45	D058-1283	BV786	BD Biosciences	Surface
	CD3	SP34-2	BUV395	BD Biosciences	Surface
	CD4	L200	BV711	BD Biosciences	Surface
	CCR5	2D5	BV605	BD Biosciences	Surface
	Live/Dead	–	Blue Dye	Thermo Fisher	Surface
B cell culture assay	CD45	D058-1283	BV786	BD Biosciences	Surface
	CD3	SP34-2	AF700	BD Biosciences	Surface
	CD19	J3-119	PE-Cy5	Beckman Coulter	Surface
	CD20	2H7	BUV395	BD Biosciences	Surface
	CD69	FN50	BV711	BD Biosciences	Surface
	CD14	M5E2	BUV805	BD Biosciences	Surface
	CD80	L307.4	BV605	BD Biosciences	Surface
	CD38	AT-1	FITC	Stemcell Technologies	Surface
	CD138	DL-101	APC-Cy7	Novus Biologicals	Surface
	Live/dead	–	Blue dye	Thermo Fisher	Surface

### Antibody Dependent Phagocytosis Assay

Antibody dependent phagocytosis (ADP) activity was determined as previously described (24, 25). Briefly, SIV_M766_ gp120 was biotinylated with a Biotin-XX Microscale Protein Labeling Kit (Life Technologies, Grand Island, NY) and incubated with a 10-fold dilution of 1 μg avidin coated sky blue fluorescent beads (0.8 μm diameter; Spherotech, Lake Forest, IL) overnight at 4°C. Enriched AMs were plated in a U-bottom 96 well plate at 40,000 cells/well and undiluted BAL-fluid (BAL-F) was added from naïve or immunized macaques. The bead-gp120 mixture was brought to a final 50-fold dilution in RPMI 1640 containing 10% FBS (R10) and 50 μl was added to the cells and incubated for 3 h at 37°C. After 3 h of incubation, 70 μl of 2% paraformaldehyde was added for fixation. Fluorescent bead uptake was assessed using a BD Biosciences LSRII flow cytometer. Bead uptake specifically by AM was made possible by focusing on cells that were autofluorescent in the fluorescein isothiocyanate (FITC) channel. The phagocytic score of each sample was calculated by multiplying the frequency of bead-positive cells by the degree of phagocytosis measured as mean fluorescence intensity (MFI) and dividing by 10^6^. For SIV-specific ADP, scores were obtained by dividing values from AM incubated with immunized BAL-F by those with naïve BAL-F. To rule out differences in macrophage activity between naïve and immunized macaques, AMs from immunized macaques incubated with PBS were used as control.

### Isolation of Alveolar Macrophages and B Cells

Six naïve macaques and an additional 6 that had received two Ad-SIV immunizations were used to assess the effect of AM on B cells. Naïve B cells were isolated from viably frozen peripheral blood mononuclear cells (PBMC) using MACS (Miltenyi Biotec). PBMC were thawed and first enriched for B cells by negative selection done by staining with anti-CD2 and anti-CD14 microbeads (non-human primate) (Miltenyi Biotec) for 15 min. The flowthrough was collected after running cells through a magnetic column. Collected cells were then stained with PE-anti-CD19 (J3-119) (Beckman Coulter) and PE-anti-CD20 (2H7) (BioLegend) antibodies for 15 min. Cells were then labeled with anti-PE magnetic beads (Miltenyi Biotec) and B cells were positively isolated using a magnetic column.

AMs obtained from BAL collected from naïve macaques or post-2^nd^ Ad immunization were sorted using a BD FACSAria™. BAL cells were stained with BV786 anti-CD45 (D058-1283), BUV395 anti-CD3 (SP34-2), APC-Cy7 anti-CD11b (ICRF44) (Thermo Fisher), and PE-Cy7 anti-CD206 (19.2). Blue LIVE/DEAD™ viability dye was used to exclude dead cells. AMs were sorted to a purity of 95%.

### B Cell Culture

AMs sorted from either naïve or immunized macaques were plated in a 48-well plate at a density of 150,000 cells per well and stimulated with 200 nM SIV_M766_ gp120 protein for 24 h. Any cells that did not attach within 1 h of plating were removed before stimulation. Supernatants were then collected and used in cultures of naïve B cells isolated as described above. B cells were resuspended in 100 µl of R10 and plated at a density of 200,000 cells/well in a 96-well U-bottom plate. B cells were cultured with an additional 100 µl of R10 as control or with supernatant from either naïve or immunized AMs. The B cells were cultured for 7 days after which the supernatant was collected and stored at −80°C for further antibody analysis. B cells were collected and stained for analysis by flow cytometry using antibodies detailed in **Table 1**. Cells were acquired using the BD FACSymphony and analyzed by FlowJo.

### Quantification of Immunoglobulins by ELISA

Total IgG antibodies in BAL-F, and total IgG, IgM, and IgA antibodies in B cell culture supernatants were measured as follows: wells of Greiner high-binding 12 area 96-well plates were coated overnight at 4°C with 50 ng/well of anti-rhesus IgG, IgM, or IgA (Alpha Diagnostics) in sodium bicarbonate buffer (pH 9.6) (Sigma-Aldrich, St. Louis, MO). Wells were blocked with 200 μl of 1% BSA diluent/blocking solution (KPL) in distilled water for 2 h at RT. BAL-F (50 μl) or B cell supernatant (50 µl) was added and incubated for 1 h at 37°C. Purified rhesus IgG, IgM, or IgA (Non-Human Primate Reagent Resource) were used to generate standard curves for total IgG, IgM, and IgA. Plates were washed five times with 1X wash solution (KPL). Horseradish peroxidase-labeled goat anti-monkey IgG (Alpha Diagnostics), anti-monkey IgA (Alpha Diagnostics), or anti-monkey IgM (Alpha Diagnostics) diluted 1:10,000 was added, and plates were incubated for 1 h at 37°C. After washing as described above, KPL 3,3’,5,5’-tetramethylbenzidene (TMB) substrate (SeraCare) was added for 10–20 min at RT. Color development was stopped with 50 μl 1 M phosphoric acid (Sigma), and plates were read at 450 nm using a BioTek plate reader. Total Ig antibodies were expressed as ng/ml of BAL-F or culture supernatant.

### Statistical Analysis

Statistical analysis was performed using one-way ANOVA with the Tukey multiple comparisons test as indicated in the figure legends. The Wilcoxon matched-pairs signed rank test was also used where indicated. Correlation analyses were performed by non-parametric Spearman correlations. Statistics were generated using GraphPad Prism.

## Results

### Cervicovaginal Macrophages Are Increased After Mucosal Ad5hr Immunization and Protein Boost

**Figure 2:**
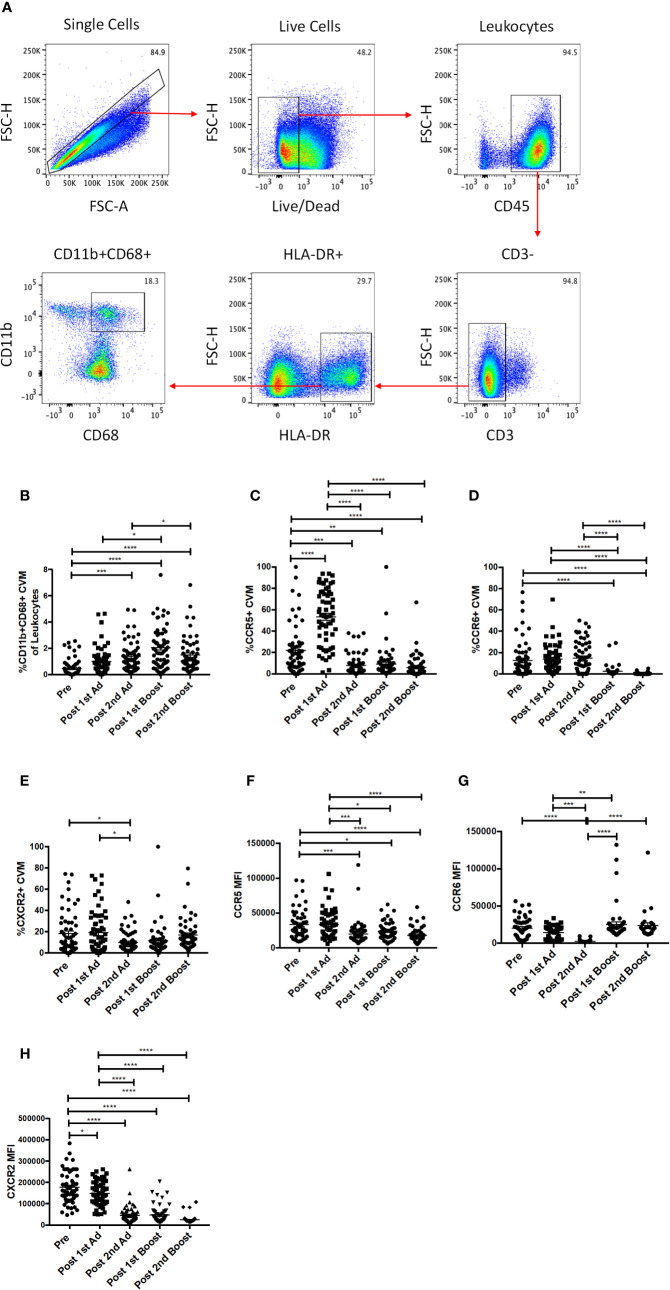
Identification and phenotyping of cervicovaginal macrophages (CVM) over the course of Immunization. **(A)** Gating strategy used to identify CVMs in cytobrush samples. Doublets and dead cells were excluded, and leukocytes were discriminated from epithelial cells by CD45 expression. CD3 expressing cells were excluded as lymphocytes. CVMs were identified as CD11b^+^HLA-DR^+^CD68^+^. **(B)** Frequency of CD11b^+^CD68^+^ CVMs in total Cytobrush leukocytes (n = 60). **(C–E)** Percentage of CVM expressing CCR5, CCR6, and CXCR2, respectively (n = 60). **(F–H)** Mean fluorescence intensity (MFI) of CCR5, CCR6, and CXCR2 expression on CVMs (n = 60). Data are represented as mean ± SEM. Statistical differences were determined using one-way ANOVA and Tukey’s multiple comparisons test. (^*^p < 0.05, ^**^p < 0.01, ^***^p < 0.001, ^****^p < 0.0001).

In order to study the responses of macrophages in the cervicovaginal mucosa, we collected cytobrushes before and 3 days after each immunization. Biopsies were not taken to avoid compromising the integrity of the mucosal surface as SIV challenge would be performed vaginally. CVM were identified as HLA-DR^+^ and CD11b^+^CD68^+^ in the live CD45^+^CD3^−^ population (**Figure 2A**). We did not find any differences in percentage of total cells, phenotype, or chemokine expression between Ad-SIV and Ad-empty groups, or SIV protein and alum groups, indicating that the changes observed could be attributed to the Ad vector and/or the alum adjuvant and not SIV antigens (data not shown). Therefore, analyses were carried out using combined vaccine and control groups. The frequency of CVM prior to immunization was <0.5% of total leukocytes in the cytobrushes (**Figure 2B**). Compared to naïve animals, the frequency significantly increased post-2^nd^ Ad and again post-1^st^ boost. In spite of a subsequent slight decrease, it remained significantly higher at the post-2^nd^ boost time point (**Figure 2B**). Chemokine receptors play an integral role in regulating immune responses (26). Binding of a chemokine ligand to its cognate receptor initiates a signaling cascade that has implications for migration and immune cell recruitment among other effects (27). We therefore wanted to assess the expression profile of these receptors on CVM over the course of immunization. There was a significant spike in the frequency of CCR5^+^ CVM post-1^st^ Ad, which dropped significantly post-2^nd^ Ad and remained significantly lower post-2^nd^ boost compared to the pre-immunization time point (**Figure 2C**). The frequency of CCR6^+^ CVM remained unchanged during priming but decreased significantly after both boosts (**Figure 2D**). The frequency of CXCR2^+^ CVM did not fluctuate dramatically but a slight significant drop in frequency was observed post-2^nd^ Ad compared to the post-1^st^ Ad and pre-vaccination time points (**Figure 2E**). The mean fluorescent intensity (MFI) of CCR5 and CXCR2 on the surface of CVM decreased over the course of immunization with a highly significant decrease in expression by the post-2^nd^ boost time point (**Figures 2F, H**). CCR6 expression tended to decrease during the Ad administrations but levels after the 2^nd^ boost were comparable to the pre-immunization time point (**Figure 2G**). Overall, the immunization strategy resulted in increased CVM frequencies when assessed 3 days after each immunization, but was not associated with increased expression of chemokine receptors at the same time point. This suggests that the immunizations may not have induced an abundance of these cell populations at the challenge site.

### Mucosal Ad5hr Administration Did Not Result in Sustained Expression of Chemokines by Cervicovaginal Macrophages

**Figure 3:**
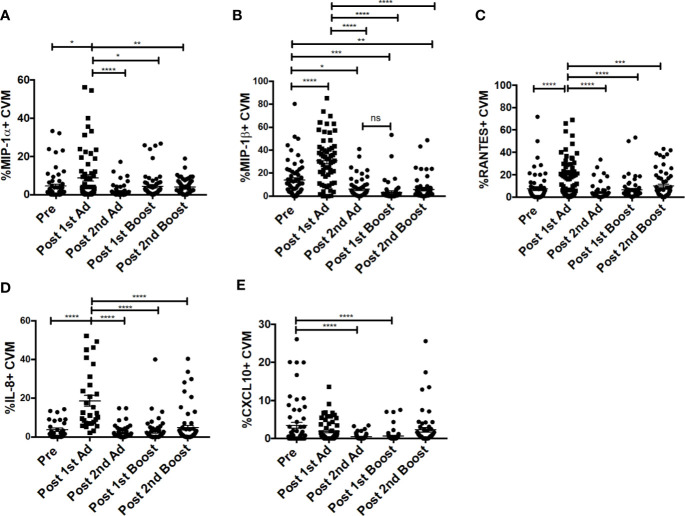
Cervicovaginal macrophages (CVM) chemokine expression peaks post-1^st^ Ad. CVM cells collected from cytobrush samples (n = 60 for all groups) were stained for intracellular expression of chemokines. **(A–E)** Percentage of CVM expressing MIP-1α, MIP-1β, RANTES, IL-8, and CXCL10, respectively. Data are represented as mean ± SEM. Statistical differences were determined using one-way ANOVA and Tukey’s multiple comparisons test. (^*^p < 0.05, ^**^p < 0.01, ^***^p < 0.001, ^****^p < 0.0001).

**Figure 4:**
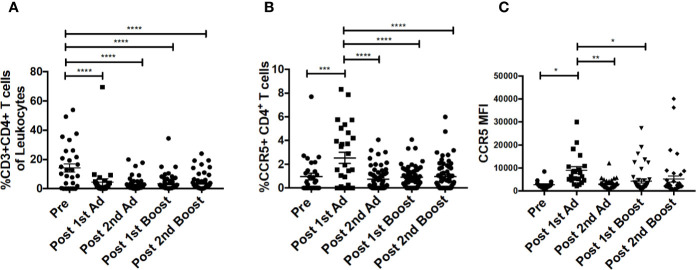
CD4^+^T cells in cytobrush samples do not increase over the course of immunization. **(A)** Frequency of CD3^+^CD4^+^ T cells in total cytobrush leukocytes. **(B)** Percentage of CD4^+^ T cells expressing CCR5 in cytobrush samples. **(C)** Expression of CCR5 on CVM CD4^+^ T cells as indicated by MFI. (n = 60 for all groups). Data are represented as mean ± SEM. Statistical differences were determined using one-way ANOVA and Tukey’s multiple comparisons test. (^*^p < 0.05, ^**^p < 0.01, ^***^p < 0.001, ^****^p < 0.0001).

Chemokines are important mediators of inflammation which play an integral role in immune surveillance, immune cell recruitment, and trafficking (27). Their expression in the vaginal mucosa has been associated with recruitment of immune cells, potentially increasing susceptibility by bringing target cells to the site of exposure (4). The Ad vector has been shown to replicate at mucosal sites irrespective of the site of administration and can target antigen presenting cells (21). Determining the chemokine responses of macrophages to the Ad at the site of challenge is important as they may have an effect on the outcome of the vaccine strategy. The CVM in the mucosa showed significantly increased frequencies of MIP-1⍺-, MIP-1β-, RANTES-, and IL-8-expressing cells post-1^st^ Ad (**Figures 3A–D**). Interestingly, the increased frequencies were limited to the post-1^st^ Ad time point as cell percentages decreased to the pre-immunization levels or below by the post-2^nd^ boost time point. The frequency of CXCL10^+^ cells did not increase post-1^st^ Ad. In fact, decreased percentages were observed post-2^nd^ Ad and post-1^st^ boost (**Figure 3E**). These results were in line with the frequency of CD4^+^ T cells which did not increase over the course of immunization (**Figure 4A**). In fact, naïve animals exhibited a higher frequency of CD4^+^ T cells than after each prime and boost administration. Despite an initial significant increase following the 1^st^ Ad prime, the frequency of CCR5^+^ cells diminished thereafter, and was comparable to levels prior to immunization (**Figure 4B**). The initial increase in frequency was supported by the increase in CCR5 MFI post-1^st^ Ad, that also diminished over the course of immunization (**Figure 4C**). These results indicate that adenoviral immunization did not create a sustained chemokine rich environment in the cervicovaginal mucosa over the course of immunization that might have resulted in CD4^+^ T cell recruitment at the time of challenge. Indeed, there might have been changes not captured at the time points chosen here for analysis; however, as innate immune cell responses occur early after immunization, it is unlikely that drastic changes occurred in the weeks between immunizations that changed the cervicovaginal environment.

### Immunization Strategy Increased Cervicovaginal Macrophage FcγRIII Expression Which Correlated With Delayed Simian Immunodeficiency Virus Acquisition

**Figure 5:**
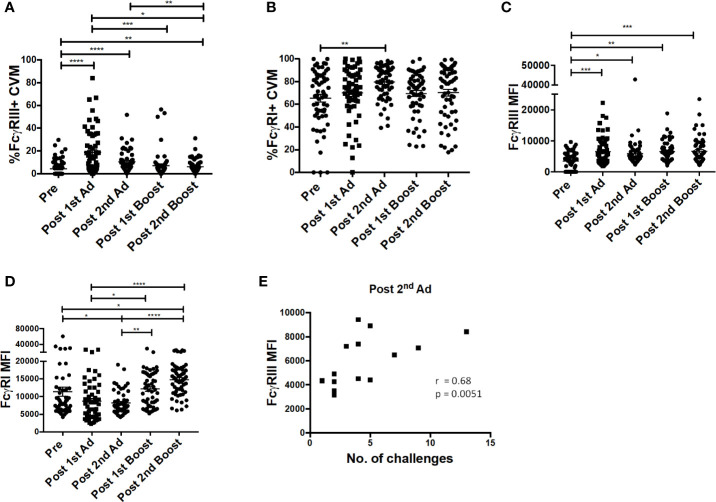
FcγR expression on cervicovaginal macrophages (CVM) is associated with simian immunodeficiency virus (SIV) acquisition delay. Cytobrushes were collected from macaques pre-immunization, post-1^st^ Ad, 2^nd^ Ad, 1^st^ boost, and 2^nd^ boost. CVM cells were recovered and stained and analyzed by flow cytometry. **(A, B)** Percentage of CVM expressing FcγRIII and FcγRI, respectively (n = 60). **(C, D)** Expression of FcγRIII and FcγRI in CVMs as indicated by MFI (n = 60). **(E)** Correlation of FcγRIII expression and number of challenges post-2^nd^ Ad (n = 16; Macaques in the Ad-control group, those treated with microbicide gel prior to challenge, and those that were protected were excluded from this correlation analysis). Data in **(A–D)**  are represented as mean ± SEM. Statistical differences were determined using one-way ANOVA and Tukey’s multiple comparisons test. (^*^p < 0.05, ^**^p < 0.01, ^***^p < 0.001, ^****^p < 0.0001). Correlation statistics were generated using Spearman correlation.

Previously, we reported a positive association between FcγRIII (CD16) expression and ADP activity by AMs from SIV-infected macaques (24). In line with this observation, we wanted to determine if our immunization strategy affected FcγR expression of macrophages at the mucosal site of challenge. Immunization increased the frequency of FcγRIII expressing CVM after the 1^st^ Ad and despite a subsequent drop, it remained significantly higher post-2^nd^ boost compared to naïve animals (**Figure 5A**). The frequency of FcγRI-expressing CVM was already high prior to immunization and remained relatively stable with a slight increase post-2^nd^ Ad (**Figure 5B**). MFI analysis showed that expression of FcγRIII, despite peaking post-1^st^ Ad as seen for the frequency, remained significantly higher during subsequent immunizations compared to the pre-immunization CVM (**Figure 5C**). Expression of FcγRI also showed a slight overall increase after the 2^nd^ boost compared to the pre-immunization time point (**Figure 5D**). In the previous study, we also found a negative association between AM FcγRIII expression and viral load (24). Interestingly, in this study we found that CVM FcγRIII expression post-2^nd^ Ad in the Ad-SIV group but not Ad-control group positively associated with the number of challenges needed for SIV acquisition (**Figure 5E**; challenge data previously reported, ref. (20)). This suggests potential protective benefits of FcγRIII expression induced by the second immunization. Indeed, the strong correlation between CVM FcγRIII expression and number of challenges in the Ad-SIV group suggests that the presence of SIV-specific antibodies may be important in mediating macrophage functions associated with delayed acquisition (**Figure 5E**). ADP and ADCC (antibody-dependent cellular cytotoxicity) are macrophage functional activities that can be mediated by FcγRs to impart protection. Our previously reported association between AM FcγRIII expression and AM-mediated SIV-specific ADP (24) suggests a similar association may exist between CVM FcγRIII expression and ADP. However, functional studies using CVM were not possible due to limited cell recovery; nevertheless a possible mechanism of protection was further explored using AM. It is however important to note that macrophages have tissue-specific phenotypes and functions, thus functional properties of AM may not apply to CVM as they represent distinct populations.

### Alveolar Macrophage Frequency Is Maintained But Cells Become Activated After Mucosal Ad5hr Immunization

**Figure 6:**
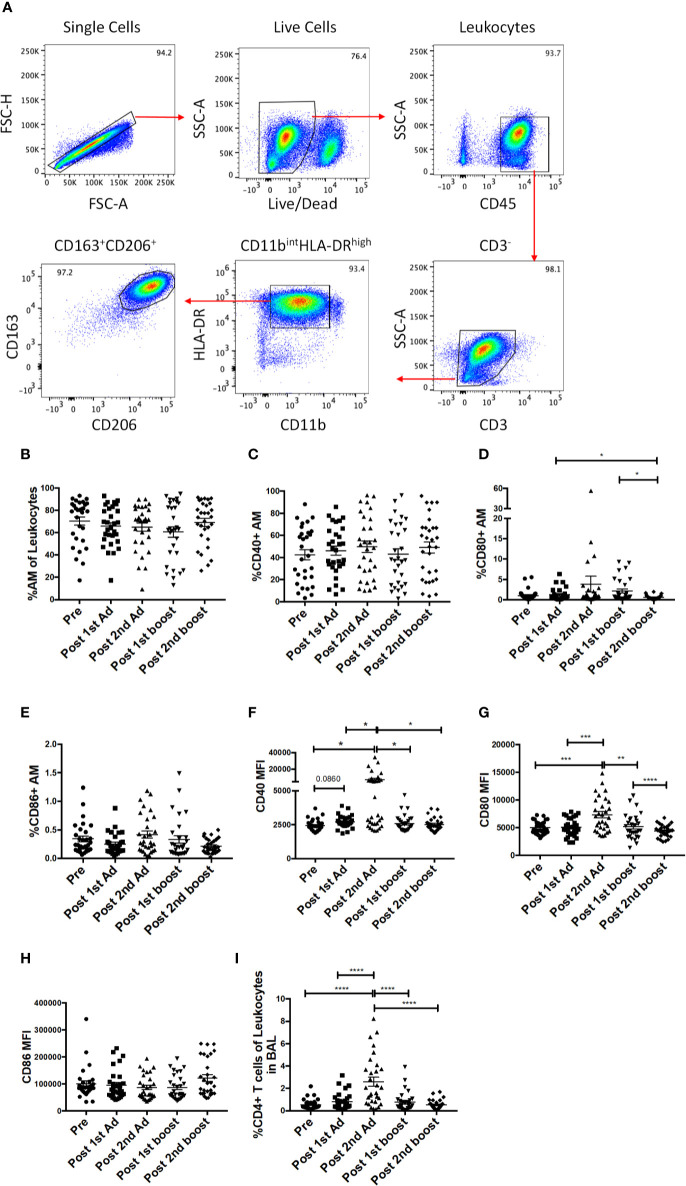
Identification of alveolar macrophages (AM) and characterization of AM activation over the course of immunization. **(A)** Gating strategy used to identify AMs in bronchoalveolar lavage (BAL) samples. Doublets and dead cells were excluded, and leukocytes were discriminated from epithelial cells by their CD45 expression. Gates were extended to include highly granular AMs. CD3 expressing cells were excluded as lymphocytes. AMs were identified as FSC^high^SSC^high^, CD11b^int^HLA-DR^high^CD163^+^CD206^+^. **(B)** Frequency of CD163^+^CD206^+^ AMs in total BAL leukocytes (n = 30 for all groups). **(C–E)** Percentage of AMs expressing CD40, CD80, and CD86, respectively. **(F–H)** Mean fluorescence intensity (MFI) of CD40, CD80, and CD86 expression on AMs. **(I)** Frequency of CD3^+^CD4^+^ T cells in total BAL leukocytes. Data are represented as mean ± SEM. Statistical differences were determined using one-way ANOVA and Tukey’s multiple comparisons test. (^*^p < 0.05, ^**^p < 0.01, ^***^p < 0.001, ^****^p < 0.0001).

As the priming immunizations were administered to the upper respiratory tract, the dynamics and function of AM in response to the vaccine regimen were of interest. AM frequencies and activation after Ad immunizations and protein boosts were evaluated using BAL samples collected at day 3 following each immunization. AM were identified by gating for CD163^+^CD206^+^ cells in the CD3^-^HLA-DR^high^CD11b^int^ leukocyte population (**Figure 6A**). The AM population was on average 70% of the total leukocyte population in BAL and did not significantly change over the course of immunization, although a slight drop in mean frequency was observed post-1^st^ boost (**Figure 6B**). Frequencies for AM also did not differ between the groups that received SIV specific antigens vs. ones which received empty Ad or alum suggesting AM responses could be attributed to the Ad and/or alum administration. CD40, CD80, and CD86 are important macrophage costimulatory and activation markers. The frequencies of AMs expressing CD40 and CD86 were not altered over all time points (**Figures 6C, E**). The frequencies of CD80^+^ AM tended to increase post-2^nd^ Ad and post-1^st^ boost compared to the pre-immunization time point but were not significant (**Figure 6D**). In general, although changes in mean AM frequencies were limited, AM activation after mucosal immunization was evident by the significantly increased expression of CD40 and CD80 after the 2^nd^ Ad prime (**Figures 6F, G**), although CD86 MFI remained unchanged over the course of immunization (**Figure 6H**). Further, we found an increased frequency of T cells in BAL by the post-2^nd^ Ad time point (**Figure 6I**). These results indicate that the Ad activated AMs and induced infiltration of CD4^+^ T cells able to interact with the antigen presenting cells.

### Adenoviral Immunization Strategy Induced Capacity of Alveolar Macrophages to Mediate Antibody Dependent Phagocytosis

**Figure 7:**
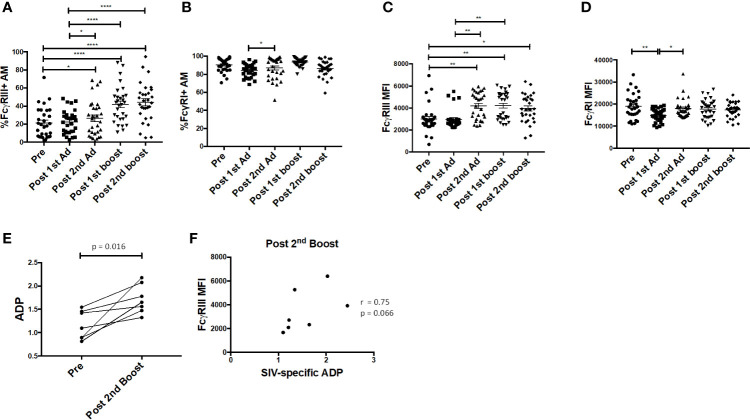
Alveolar macrophages (AM) FcγR expression and phagocytic activity. **(A**, **B)** Percentage of AM in bronchoalveolar lavage (BAL) samples (n = 30) expressing FcγRIII and FcγRI, respectively. **(C**, **D)** Mean fluorescence intensity (MFI) of FcγRIII and FcγRI expression in AMs. **(E)** Antibody dependent phagocytosis (ADP) activity in the presence of autologous BAL fluid (BAL-F) was assayed using AMs from seven macaques taken pre-immunization and post-2^nd^ boost (n = 7). **(F)** SIV-specific activity was calculated by normalizing phagocytosis activity post-2^nd^ boost to pre-immunization conditions, where SIV specific antibodies were absent in the BAL-F. Correlation of AM FcγRIII expression with SIV-specific ADP (n = 7). Data in **(A**–**D)** are represented as mean ± SEM. Statistical differences were determined using one-way ANOVA and Tukey’s multiple comparisons test. **(A**–**D)** (^*^p < 0.05, ^**^p < 0.01, ^****^p < 0.0001). Correlation statistics were generated using Spearman correlation.

Fcγ receptors are expressed on AMs and importantly are needed for phagocytosis of opsonized antigens (28). Here, the frequency of FcγRIII^+^ AMs was significantly enhanced post-2^nd^ Ad and continued to increase after the second boost, while the frequency of FcγRI^+^ AM remained constant (**Figures 7A, B**). The MFI of FcγRIII was also increased after the 2^nd^ Ad immunization and remained increased after the 2^nd^ boost (**Figure 7C**). The AM FcγRI expression level remained high and did not show drastic changes in response to the immunization regimen, despite a slight drop after the 1^st^ Ad which returned to the pre-immunization level after the 2^nd^ Ad (**Figure 7D**). We have previously shown that AM from SIV-infected macaques are capable of mediating ADP in the presence of SIV-specific antibodies (24). To assess this functional activity of AMs after immunization, we looked at the ability of AMs to phagocytose SIV_M766_ gp120-coated beads in the presence of autologous BAL-F. Upon immunization, the ability of AM to mediate ADP was enhanced as seen by the significant increase in phagocytosis score post-2^nd^ boost (**Figure 7E**). SIV-specific ADP activity was obtained by normalizing the phagocytosis activity post-2^nd^ boost to that obtained from pre-immunization conditions, where SIV-specific antibodies were absent in the BAL-F. Upon normalization, SIV-specific ADP positively correlated with FcγRIII expression at the post-2^nd^ boost time point (**Figure 7F**) in accordance with our previous study (24). A positive association between increased FcγRIII receptor and ADP underscores the functional importance of inducing this receptor.

### Alveolar Macrophages Show Potential to Mediate B Cell Help

**Figure 8:**
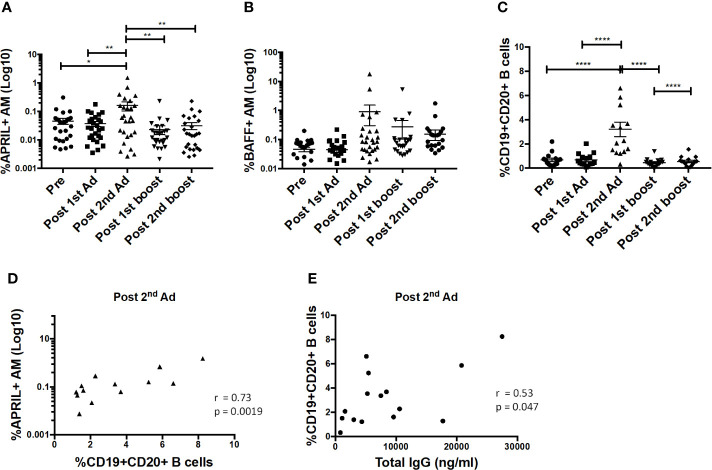
Alveolar macrophages (AM) show increased a proliferation-inducing ligand (APRIL) expression post-2^nd^ Ad. AM cells collected from bronchoalveolar lavage (BAL) samples were stained for intracellular expression of B cell-activating factor (BAFF) and APRIL. Percentage of AMs (n = 30) expressing **(A)** APRIL and **(B)** BAFF. **(C)** Frequency of CD19^+^CD20^+^ B cells of total leukocytes in BAL (n = 15). **(D)** Correlation of APRIL expressing AMs with frequency of B cells in BAL (n = 15). **(E)** Correlation of B cell frequency in BAL with total IgG in bronchoalveolar lavage fluid (BAL-F) (n = 15). **(A**–**C)** Data are represented as mean ± SEM. Statistical differences were determined using one-way ANOVA and Tukey’s multiple comparisons test. (^*^p < 0.05, ^**^p < 0.01, ^****^p < 0.0001). **(D**, **E)** Correlation statistics were generated using Spearman correlation.

B cell-activating factor (BAFF) and a proliferation-inducing ligand (APRIL) are important cytokines that regulate B cell activation, induce proliferation, and increase cell survival [reviewed in (29)]. Interaction of these cytokines with receptors on B cells has been shown to lead to increased cell survival and antibody-isotype switching (30). Here, the frequency of APRIL-expressing AMs significantly increased after the 2^nd^ Ad immunization (**Figure 8A**). The frequency of BAFF-expressing AM also tended to increase at the same time point, although not significantly (**Figure 8B**). The B cell frequency in BAL also significantly increased after the 2^nd^ Ad administration (**Figure 8C**). Similar to overall macrophage frequencies, there were no differences in frequencies of AM expressing cytokines or total B cells when comparing the Ad-SIV and control Ad-empty groups, or SIV-protein and Alum only groups. To investigate the connection between cytokine expressing AMs and B cells, we performed correlation analyses. Indeed, the frequency of APRIL^+^ AMs positively correlated with the B cell frequency in BAL at the post-2^nd^ Ad time point (**Figure 8D**). Further, the B cell frequency correlated with the total IgG in BAL-F post-2^nd^ Ad highlighting the association between AM APRIL expression and B cell activation (**Figure 8E**).

**Figure 9:**
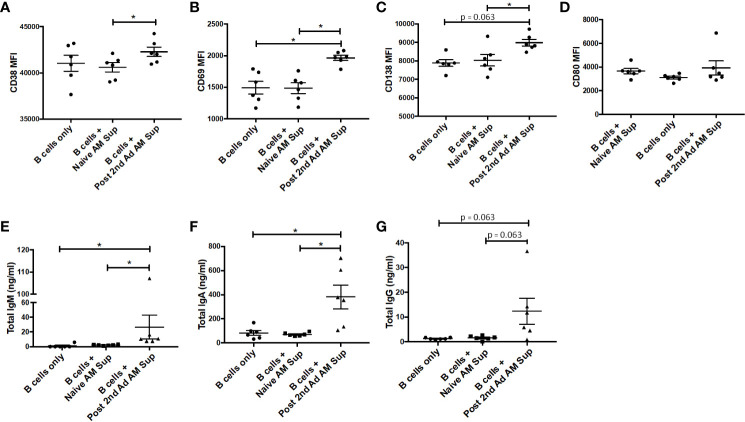
Effect of alveolar macrophage (AM) factors on B cell activation and antibody secretion. AMs from six macaques taken prior to immunization or post-2^nd^ Ad were sorted by fluorescence-activated cell sorting (FACS) and cultured for 24 h in the presence of simian immunodeficiency virus (SIV) gp120. Naïve B cells isolated from peripheral blood mononuclear cells (PBMCs) of the macaques were cultured for 7 days with fresh R-10 or supernatant from the AMs and stained for analysis by flow cytometry. Mean fluorescent intensity (MFI) of **(A)** CD38, **(B)** CD69, **(C)** CD138, **(D)** CD80. Total **(E)** IgM, **(F)** IgA, **(G)** IgG levels in supernatant derived from 7-day B cell culture. Data are represented as mean ± SEM. Statistical differences were determined using Wilcoxon paired test. (^*^p < 0.05).

In order to further characterize effects that soluble factors released by AMs may have on B cells, we performed a B cell culture assay using AM supernatant. AMs from naïve or immunized macaques at the post-2^nd^ Ad time point were sorted by FACS to a purity of 95% and cultured for 24 h in the presence of gp120 to stimulate cytokine production. PBMCs derived from naïve macaques were enriched for B cells and then cultured with fresh media only or with supernatant from the stimulated AMs for 7 days. There was a clear significant induction in expression of the plasma cell differentiation marker CD38 and the activation marker CD69 on B cells cultured with AM supernatant from immunized macaques, compared to those from naïve macaques (**Figures 9A, B**). A significant difference in CD69 expression was also evident when comparing the immunized group with the media only B cell culture further underscoring the role AMs were playing in the B cell activation observed. Such a difference was not seen in the case of CD38 expression, although the mean remained higher in the immunized group (**Figure 9A**). B cells cultured with immunized AM supernatant also showed increased CD138 expression suggesting the B cells were differentiating into plasma cells (31) (**Figure 9C**). No difference in CD80 expression was observed among all groups (**Figure 9D**). B cells cultured with AM supernatant from immunized macaques showed significantly higher levels of IgM and IgA compared to B cells cultured with naïve AM supernatant or B cells only, while the IgG levels in B cells cultured with AM supernatant showed similar trends (**Figures 9E–G**). Taken together, the data indicate B cells can be influenced by AMs leading to their activation and differentiation indicative of B cell help.

## Discussion

The epithelium lining the cervicovaginal compartment is the first line of defense against HIV entry during vaginal exposure as it provides a physical and immunological barrier. Therefore, candidate vaccines need to elicit strong mucosal immune responses to prevent infection. Ad vaccine vectors have been shown to replicate at mucosal sites making them targets for innate immune cells and therefore inducing activation (21). On the one hand, this can be beneficial as recognition of pathogen-associated molecular patterns (PAMPS) can lead to release of chemokines and cytokines that mediate inflammation and initiate innate immune responses (32). Further, chemokines like MIP-1⍺, MIP-1β, and RANTES can bind CCR5 and block CCR5-based viral entry (33). However, when it comes to HIV, inflammatory responses can attract CD4^+^ T cells to the site of exposure. Here, CVM activation was observed after the 1^st^ Ad and suggests a potential impact on exposure outcome at that time point. Beyond the initial activation, our vaccine strategy did not create a sustained inflammatory environment at the vaginal mucosa that would be conducive to acquiring SIV. Moreover, we found that the vaccine regimen led to increased FcγRIII expression on CVM which was associated with delayed acquisition. We also found increased AM activation, induction of ADP activity, and positive associations between B cells and APRIL expressing AMs in BAL, indicating strong vaccine-induced responses with potentially beneficial effects at both mucosal sites.

We did not find differences in macrophage frequencies or chemokine profiles between Ad-SIV and Ad-empty, or between SIV gp120 in alum and alum only groups indicating that any responses observed resulted from stimulation by the replicating adenovirus acting as an adjuvant or the alum. Increased frequency of CCR5^+^ CVM was observed after the first Ad immunization; however, expression of CCR5, CCR6, and CXCR2 on CVM diminished over the course of immunization (**Figures 2F–H**). Downregulation of CCR5 expression by CC-chemokines that bind to the receptor has been reported (34). In fact, we observed an increase in expression of MIP-1⍺, MIP-1β, and RANTES after the 1^st^ Ad (**Figures 3A–C**) which may have subsequently led to reduced expression of CCR5. We also observed increased IL-8 expression post-1^st^ Ad (**Figure 3D**), although no such inverse association between IL-8 and its receptor CXCR2 has been reported. In general, as CCR5^+^ macrophages can also act as targets for viral entry (35), lower frequency of this cell population at the site of challenge is beneficial. More recent studies have shown that CD4^+^ T cells are preferential targets of HIV/SIV for entry after vaginal exposure (2, 5). However, we did not find significant increases in the total CD4^+^ T cell population that might have impacted susceptibility (**Figure 4A**).

Our immunization strategy resulted in increased expression of FcγRIII on CVM after the 2^nd^ Ad immunization, which correlated with delayed acquisition (**Figures 5C, E**). Although the challenge was performed 32 weeks after this last Ad administration, there was a continuous increase in expression of FcγRIII past the 2^nd^ boost time point. This sustained increase in expression may well be associated with the Ad administration. Patterson *et al*. demonstrated significant presence of replicating Ad in macrophages of rectal tissues at least 25 weeks after an Ad immunization (21). It is possible that the replicating Ad used in this study also persisted in the vaginal mucosa, and thus continued to impact the challenge outcome after the 2^nd^ boost time point. Since sufficient CVM were not available for functional assays, while exploring possible mechanisms of action using AM, we found a correlation between AM FcγRIII expression and ADP post-2^nd^ boost (**Figure 7F**). The fact that the CVM FcγRIII correlation with delayed acquisition was observed in the Ad-SIV group implies that such antibody dependent function may be contributing to protection. A previous study that assessed the efficacy of an Ad-based vaccine in combination with an Env boost against SIV infection in rhesus macaques found correlations between ADCP, ADCC, and protection (36). Other studies have shown that FcγRIII is important for mediating ADCC by monocytes (37). Whether the delayed acquisition we observed was directly associated with FcγRIII-mediated ADP or ADCC remains to be elucidated. However, the results highlight the importance of inducing expression of FcγRIII by macrophages at the site of virus exposure.

AM are well positioned to directly encounter the Ad immunogens administered intranasally and intratracheally. As important regulators of lung homeostasis, AMs need to maintain plasticity in their function so as to quickly recognize PAMPS and protect the host but also not initiate an inflammatory response for every foreign antigen encountered *via* the airway [reviewed in (38)]. It has been suggested that reduced inflammatory responses by AMs is maintained partly by decreased expression of the costimulatory molecules CD80 and CD86, required for T cell activation (39). Here, we found significant increases in the expression of the CD40 and CD80 on AMs after the 2^nd^ Ad administration (**Figures 6F, G**). In keeping with this, we also found a significantly increased percentage of CD4^+^ T cells (**Figure 6B**) and CD19^+^CD20^+^ B cells in BAL post-2^nd^ Ad (**Figure 8C**). This suggests that Ad can be a potent stimulator of AMs leading to increased expression of costimulatory molecules and ability to efficiently present antigen to T cells as well as express cytokines that can induce B cell proliferation.

A direct role for macrophages in activating B cells has been shown in the context of delivering and accumulating antigen, particularly those with larger molecular weights and virus particulates, in the lymph node subcapsular sinus (40, 41). This brings macrophages and antigen carrying macrophages in close proximity to B cells in the follicles. In studies that included removal of macrophages using clodronate, lack of local B cell activation and systemic viral dissemination was observed (41–43). As migration of AMs to lung dLNs has been reported (17), it is possible that AMs are migrating to the lung dLNs in response to the adenoviral immunization resulting in the local B cell expansion observed in BAL, either directly or indirectly. In our study, we observed increased activation of B cells when cultured with SIV Env-stimulated AM supernatants from immunized macaques (**Figure 9**), the same time point at which we observed significantly increased APRIL^+^ AM and total B cell frequencies (**Figures 8A, C**). A functional role for cytokines released by AMs in activating B cells in a vaccine setting has not been reported before. Previous studies have reported a role for APRIL in B cell survival, proliferation and differentiation in healthy bone marrow (44–47). In addition, in disease settings such as immunoglobulin G4-related disease, APRIL producing macrophages have been shown to infiltrate plasma cell-rich lesions, with secreted APRIL sustaining the plasma cells in the lesions and maintaining disease presentation (48). Here, differentiation into plasma cells in response to AM secreted factors was suggested by increased expression of CD38 and CD138 (**Figures 9A, C**). There was also a clear indication of an activated B cell population as seen by increased CD69 expression (**Figure 9B**). Increased B cell activation in the presence of vaccinated AM supernatant further translated to higher levels of total IgA, IgM, and, although not significant, IgG in the B cell culture supernatant (**Figures 9E–G**). Even though no differences were seen in total antibody production between B cells alone and B cells cultured with naïve AM supernatant, IgA antibody levels were higher than those observed for IgM or IgG. One explanation for this could be the presence of B-1 cells that have been shown to spontaneously secrete Ig in rhesus macaques (49). Particularly, in humans, a population of B-1 cells with a pre-plasmablast phenotype in PBMC have been shown to spontaneously secrete IgA (50), suggesting that similar antibody secretion may be responsible for the higher IgA levels observed here. Nevertheless, clear differences were seen between vaccinated and unvaccinated AM supernatant and B cell culture settings indicating that AM can provide favorable conditions for increased B cell antibody production.

When comparing CVM and AM responses to the Ad vector, significant changes were observed mostly after the 1^st^ Ad for CVMs, while AM responses were limited to post-2^nd^ Ad. However, a direct comparison could not be made as different markers were assessed. Chemokines and chemokine receptors appear to be upregulated by CVMs upon initial Ad encounter but diminished during subsequent immunizations. A contributing factor could be the presence of Ad-specific antibodies generated after the 1^st^ Ad administration, which could blunt immune responses to the 2^nd^ Ad immunization. However, this would not explain the responses seen in AM. The fact that significant changes in AM activation were found after the 2^nd^ Ad administration might be due to a phenomenon described as trained innate immunity. In trained immunity, encounter of innate immune cells by a pathogen either during infection or vaccination, leads to epigenetic changes that enhance their response during a repeated exposure [reviewed in (51)]. This might be relevant particularly to AMs as quick immune responses to antigens in the lungs are not desirable and further suggests differences between CVM and AM which remain to be discovered. It is also possible that the different kinetics of the immune responses seen in CVM and AM reflect the differences in how these distinct macrophage populations are affected by mucosal vaccination routes. Of note, FcγRIII expression was induced after the 1^st^ Ad in CVM whereas changes in FcγRIII expressing AM were documented only after the 2^nd^ Ad. The first Ad immunization was administered orally and intranasally. Nasal immunization in particular has been shown to stimulate immunity in the rectal/genital tract (52). The second Ad administration was given intratracheally, and presumably was more effective in stimulating immune responses in the lungs.

Taken together, our results show that the mucosal replicating Ad5hr immunizations and systemic Env boosting recruited FcγRIII^+^ macrophages to the cervicovaginal compartment, which strongly associated with delayed acquisition. Further, our findings identify a role for the Ad immunization strategy that enhances AM antigen presentation to T cells and ability to provide B cell help. The contribution of macrophages to adaptive immune responses and the protective capacity of FcγRIII expression by macrophages is valuable information that can be harnessed for use in future vaccine studies.

## Data Availability Statement

All datasets presented in this study are included in the article/supplementary material.

## Ethics Statement

The animal study was reviewed and approved by the NCI Animal Care and Use Committee.

## Author Contributions

RH and MR-G: conceptualization. RH, SH, and MR-G: methodology. RH, SH, CH, MR, and GE-A: investigation. RH, SH, and TH: resources. RH: writing—original draft. RH, SH, GE-A, MR, CH, EK, TH, and MR-G: writing—review and editing. MR-G: supervision. All authors contributed to the article and approved the submitted version.

## Funding

This work was funded by the Intramural Research Program of the National Institutes of Health, National Cancer Institute.

## Conflict of Interest

The authors declare that the research was conducted in the absence of any commercial or financial relationships that could be construed as a potential conflict of interest.
